# Loss of LRPPRC causes ATP synthase deficiency

**DOI:** 10.1093/hmg/ddt652

**Published:** 2014-01-06

**Authors:** Arnaud Mourier, Benedetta Ruzzenente, Tobias Brandt, Werner Kühlbrandt, Nils-Göran Larsson

**Affiliations:** 1 Department of Mitochondrial Biology, Max Planck Institute for Biology of Ageing, Joseph-Stelzmann-Strasse 9b, Cologne 50931, Germany and; 2 Department of Structural Biology, Max Planck Institute of Biophysics, Max-von-Laue-Str. 3, Frankfurt am Main 60438, Germany

## Abstract

Defects of the oxidative phosphorylation system, in particular of cytochrome-*c* oxidase (COX, respiratory chain complex IV), are common causes of Leigh syndrome (LS), which is a rare neurodegenerative disorder with severe progressive neurological symptoms that usually present during infancy or early childhood. The COX-deficient form of LS is commonly caused by mutations in genes encoding COX assembly factors, e.g. SURF1, SCO1, SCO2 or COX10. However, other mutations affecting genes that encode proteins not directly involved in COX assembly can also cause LS. The leucine-rich pentatricopeptide repeat containing protein (LRPPRC) regulates mRNA stability, polyadenylation and coordinates mitochondrial translation. In humans, mutations in *Lrpprc* cause the French Canadian type of LS. Despite the finding that LRPPRC deficiency affects the stability of most mitochondrial mRNAs, its pathophysiological effect has mainly been attributed to COX deficiency. Surprisingly, we show here that the impaired mitochondrial respiration and reduced ATP production observed in *Lrpprc* conditional knockout mouse hearts is caused by an ATP synthase deficiency. Furthermore, the appearance of inactive subassembled ATP synthase complexes causes hyperpolarization and increases mitochondrial reactive oxygen species production. Our findings shed important new light on the bioenergetic consequences of the loss of LRPPRC in cardiac mitochondria.

## INTRODUCTION

Leigh syndrome (LS, MIM 256000) is a genetically and biochemically heterogeneous neurodegenerative entity characterized by subacute necrotizing encephalopathy predominantly affecting the thalamus, brain stem and spinal cord (1). LS patients typically develop sudden onset of severe brain stem dysfunction, psychomotor regression, hypotonia, ataxia, and lactic acidosis during their infancy or early childhood and display various types of biochemical defects, including deficient oxidative phosphorylation (2). The oxidative phosphorylation system (OXPHOS) is located in the mitochondrial inner membrane and is composed of two functional entities, i.e. the respiratory chain (RC) and the phosphorylation system, which includes the ATP synthase and carriers, such as the ATP/ADP carrier (ANT) and the phosphate carrier (PiC). The RC is historically defined as consisting of four complexes, denoted complex I–IV, which perform substrate oxidation to drive proton extrusion from the mitochondrial matrix to the intermembrane space. The proton electrochemical potential across the inner mitochondrial membrane (ΔΨ) is used by the ATP synthase to drive ATP synthesis thus coupling proton transport to ATP production.

Whereas various types of defects in oxidative phosphorylation or the pyruvate dehydrogenase enzyme complex can cause LS (3–7), complex IV (cytochrome-*c* oxidase, COX) deficiency is one of the most common causes. Mutations in various nuclear genes have been reported in COX-deficient LS, e.g. in *Surf1* (8,9), *Sco1* (10), *Sco2* (11), *Cox10* (12) and *Cox15* (13), and these genes all encode proteins involved in assembly or biogenesis of COX. Surprisingly, *Surf1* knockout (14,15) and *Sco2* knock-in (16) mouse models appear healthy and may even have increased lifespan despite a profound COX deficiency (17,18).

An unusual COX-deficient form of LS, the French Canadian type of LS (LSFC, MIM 220111), has been shown to be caused by mutations in the gene for the leucine-rich pentatricopeptide repeat containing protein (*Lrpprc*) (19), which is a key posttranscriptional regulator of mtDNA expression (20). In comparison with other forms of COX-deficient LS, patients with LSFC seem to have more severe symptoms associated with an acute metabolic crisis leading to high mortality at early ages (21,22). We have previously generated whole body *Lrpprc* knockout mice and demonstrated that this condition causes embryonic lethality (20), in agreement with results from an independent knockout strain (23). Furthermore, we found that tissue-specific inactivation of *Lrpprc* in the heart causes a progressive cardiomyopathy resulting in death before the age of 16 weeks (20). Studies of RNAi-knockdown cell lines have shown that LRPPRC is involved in regulating the stability of mtDNA-encoded mRNAs (24,25). However, the *in vivo* function of LRPPRC is not restricted to regulating mRNA stability because mice with conditional knockout of *Lrpprc* and fruit flies with knockdown of *Bsf,* the fly homolog of *Lrpprc* a.k.a. *DmLrpprc1*, have additional strong biochemical phenotypes, i.e. impaired polyadenylation of mtDNA-encoded mRNAs and loss of mitochondrial translation coordination (20,26). Intriguingly, unlike the COX-deficient mice mentioned above, the *Lrpprc* heart-specific knockout mice develop a lethal cardiomyopathy, suggesting that COX deficiency may not provide the sole explanation of the phenotype.

In this study, we decided to carefully characterize the bioenergetic properties of *Lrpprc* knockout heart mitochondria. Unexpectedly, we show that the COX deficiency *per se* cannot explain the severe OXPHOS dysfunction observed in *Lrpprc* heart knockout mice. Instead, we show that LRPPRC deficiency is associated with an ATP synthase assembly defect leading to a drastic loss of function of this enzyme. Our work thus shows that the bioenergetic defect in *Lrpprc* knockout heart mitochondria is not driven by COX deficiency but rather caused by an ATP synthase deficiency.

## RESULTS

### Loss of LRPPRC induces defective respiration under phosphorylating conditions

**Figure 1.**
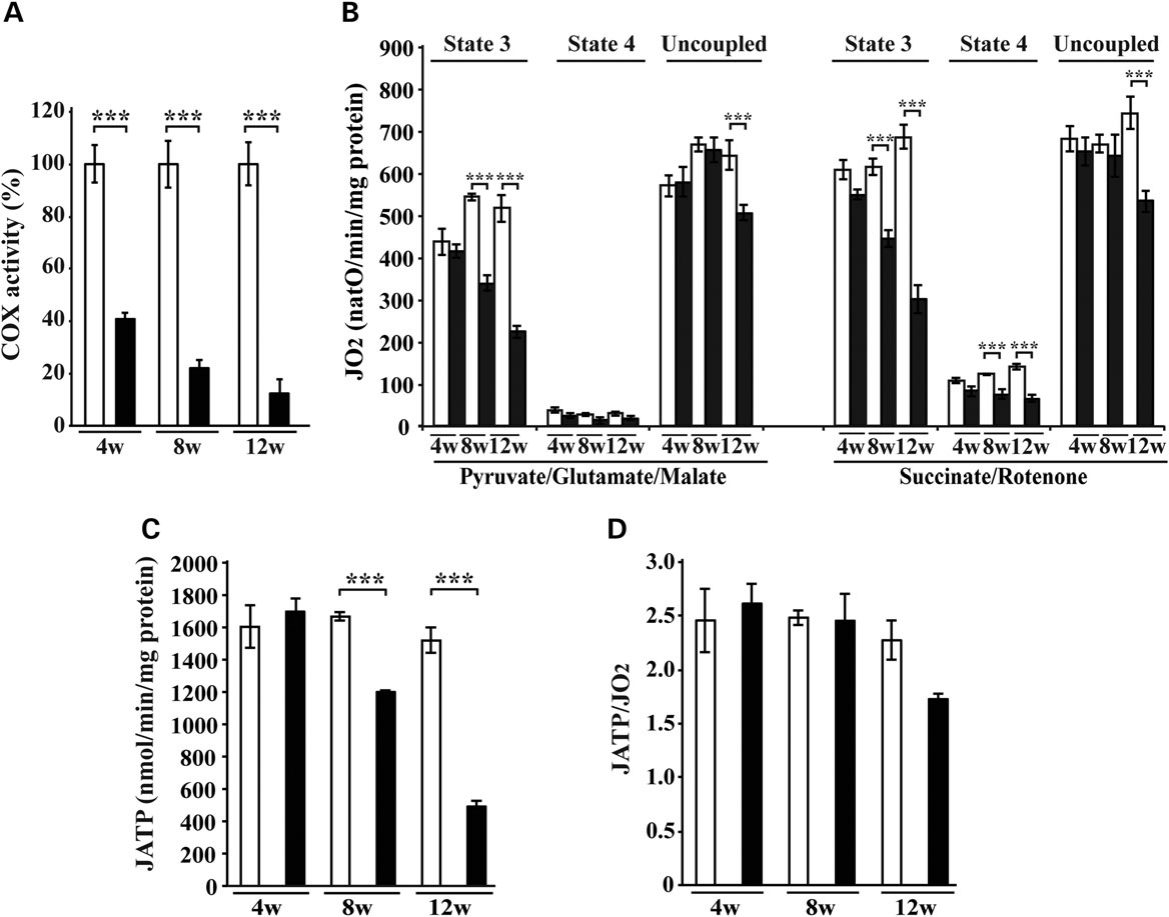
Loss of LRPPRC results in OXPHOS dysfunction. (**A**) The COX enzyme activity was measured in heart mitochondria from *Lrpprc* knockout and control at age 4 weeks (4w), 8 weeks (8w) and 12 weeks (12w). Open bars, controls (*n* = 4); filled bars, knockouts (*n* = 4). Error bars indicate mean ± SEM (^*^P < 0.05; ^**^P < 0.01; ^***^P < 0.001). (**B**) Oxygen consumption of heart mitochondria from *Lrpprc* knockout and control at different ages. Isolated mitochondria were incubated with substrates feeding electrons to complex I (pyruvate, glutamate, malate) or complex II (succinate combined with complex I inhibition by rotenone). Each set of substrates was successively combined with ADP (to assess the phosphorylating respiration: state 3), oligomycin (to measure the non-phosphorylating respiration: state 4) and finally uncoupled by adding an increasing concentration of CCCP. Open bars, control [*n* = 4 (at age 4 and 8 weeks) *n* = 15 (at age 12 weeks)]; filled bars, knockout [*n* = 4 (at age 4 and 8 weeks) *n* = 15 (at age 12 weeks)]. Error bars indicate means ± SEM (^*^P < 0.05; ^**^P < 0.01; ^***^P < 0.001). (**C**) ATP synthesis flux assessed in heart mitochondria from *Lrpprc* knockout and control at the age of 12 weeks, in the presence of succinate and rotenone. Open bars, control (*n* = 3–4); filled bars, knockout (*n* = 3–4). Error bars indicate means ± SEM (^*^P < 0.05; ^**^P < 0.01; ^***^P < 0.001). (**D**) Measurement of the oxidative phosphorylation coupling yield (nmol ATP/natO) in heart mitochondria from *Lrpprc* knockout and controls at different ages. Open bars, control (*n* = 4); filled bars, knockout (*n* = 4). Error bars indicate means ± SEM (^*^P < 0.05; ^**^P < 0.01; ^***^P < 0.001).

We assessed COX activity in conditional *Lrpprc* knockout mouse hearts and found a progressive deficiency (Fig. 1A), consistent with our previous results and reports of severe COX deficiency in LSFC patients (20). The COX deficiency in *Lrpprc* knockout hearts was profound with 40% remaining activity at age 4 weeks and 10% remaining activity at age 12 weeks (Fig. 1A). We proceeded to investigate the bioenergetic consequences of the strong decrease in COX activity by assessing the oxygen consumption rate in freshly isolated cardiac mitochondria (Fig. 1B). Mitochondria were incubated with respiratory substrates whose metabolism result in delivery of electrons at the level of complex I (pyruvate, glutamate, malate) or complex II (succinate + rotenone) and the oxygen consumption rate was recorded in the phosphorylating (state 3: ADP and Pi), non-phosphorylating (state 4: oligomycin to inhibit ATP synthase) and uncoupled state (uncoupling agent CCCP). Interestingly, the respiration in *Lrpprc* heart knockout mitochondria was profoundly affected in the phosphorylating state, whereas uncoupled respiration was either unaffected or only mildly affected at the latest studied time point (Fig. 1B). The ATP production rate was assessed in the presence of succinate, rotenone and ADP, as previously described (27), and was normal in *Lrpprc* heart knockout mitochondria at the age of 4 weeks, and strongly impaired at the ages of 8 and 12 weeks (Fig. 1C). We also assessed ATP production rate with pyruvate, glutamate, malate and ADP, and found a strong impairment in *Lrpprc* heart knockout mitochondria at the age of 12 weeks (Supplementary Material, Fig. S1A). The coupling yield of the oxidative phosphorylation, i.e. the ratio between ATP production and oxygen consumption (JATP/JO_2_), was normal at all studied ages (Fig. 1D). In summary, these findings show that the massive reduction in COX activity (Fig. 1A) is associated with an impaired respiration in the phosphorylating state (Fig. 1B) and reduced ATP synthesis (Fig. 1C, Supplementary Material, Fig. S1A), which all occur concomitantly with normal coupling of the oxidative phosphorylation system in mitochondria lacking LRPPRC (Fig. 1D). Surprisingly, the overall capacity of the RC was only mildly affected in the uncoupled state in the absence of LRPPRC (Fig. 1B).

### The respiratory defect in the phosphorylating state is not caused by the COX deficiency

**Figure 2.**
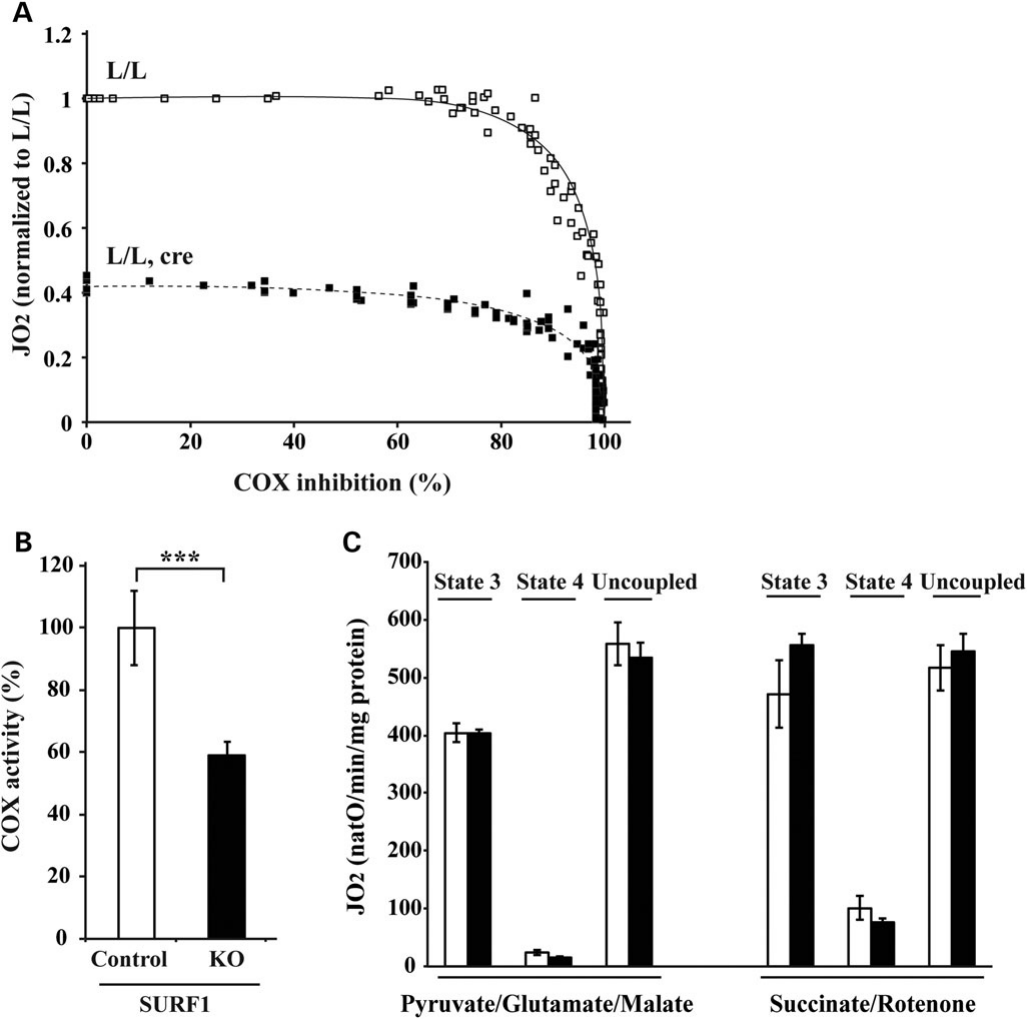
COX deficiency does not explain the mitochondrial respiration deficiency in phosphorylating conditions. (**A**) Threshold curves were performed on heart mitochondria from *Lrpprc* knockout and control mice at the age of 12 weeks in the presence of succinate, rotenone and ADP. Open square, control (*n* = 4); filled square, knockout (*n* = 4). (**B**) COX enzyme activity was measured in heart mitochondria from *Surf1* knockout and control mice at the age of 40 weeks. Open bars, control (*n* = 4); filled bars, knockout (*n* = 4). Error bars indicate means ± SEM (^*^P < 0.05; ^**^P < 0.01; ^***^P < 0.001). (**C**) Oxygen consumption was assessed in heart mitochondria from *Surf1* knockout mice at age 40 weeks. Open bars, control (*n* = 4); filled bars, knockout (*n* = 4). Error bars indicate means ± SEM (^*^P < 0.05; ^**^P < 0.01; ^***^P < 0.001).

We were intrigued by the finding that loss of ∼90% of the COX enzyme activity only mildly impaired the RC capacity and therefore we proceeded to analyze to what extent inhibition of COX reduces respiration under phosphorylating conditions with the substrates succinate, rotenone and ADP (Fig. 2A) or pyruvate, glutamate, malate and ADP (Supplementary Material, Fig. S1B). We progressively inhibited COX by cyanide treatment of wild-type cardiac mitochondria and found that the respiratory rate (JO_2_) in the phosphorylating state is only mildly altered until COX is inhibited so that only <10% of the original activity remains (Fig. 2A, Supplementary Material, Fig. S1B). To validate these conclusions by an independent set of experiments, we studied respiration in mitochondria isolated from hearts of *Surf1* knockout mice (Fig. 2B and C). The COX activity in heart mitochondria from 40-week-old *Surf1* knockout mice is reduced by ∼40% (Fig. 2B), but the respiration is unaffected (Fig. 2C), consistent with the absence of cardiomyopathy in these knockout mice (14). Next, we progressively inhibited the residual COX activity by cyanide treatment of heart mitochondria from conditional *Lrpprc* knockout mice at age 12 weeks (residual COX activity ∼10%; Fig. 1A) and found no further impairment of respiration until <60% of the residual COX activity remained (corresponding ∼6% of the COX activity in wild-type heart mitochondria) (Fig. 2A, Supplementary Material, Fig. S1B). These results provide additional support for the conclusion that the severe reduction of respiration in the phosphorylating state in heart mitochondria from conditional *Lrpprc* knockout mice is not explained by the profound COX deficiency.

### Loss of LRPPRC impairs assembly and enzyme activity of the ATP synthase

**Figure 3.**
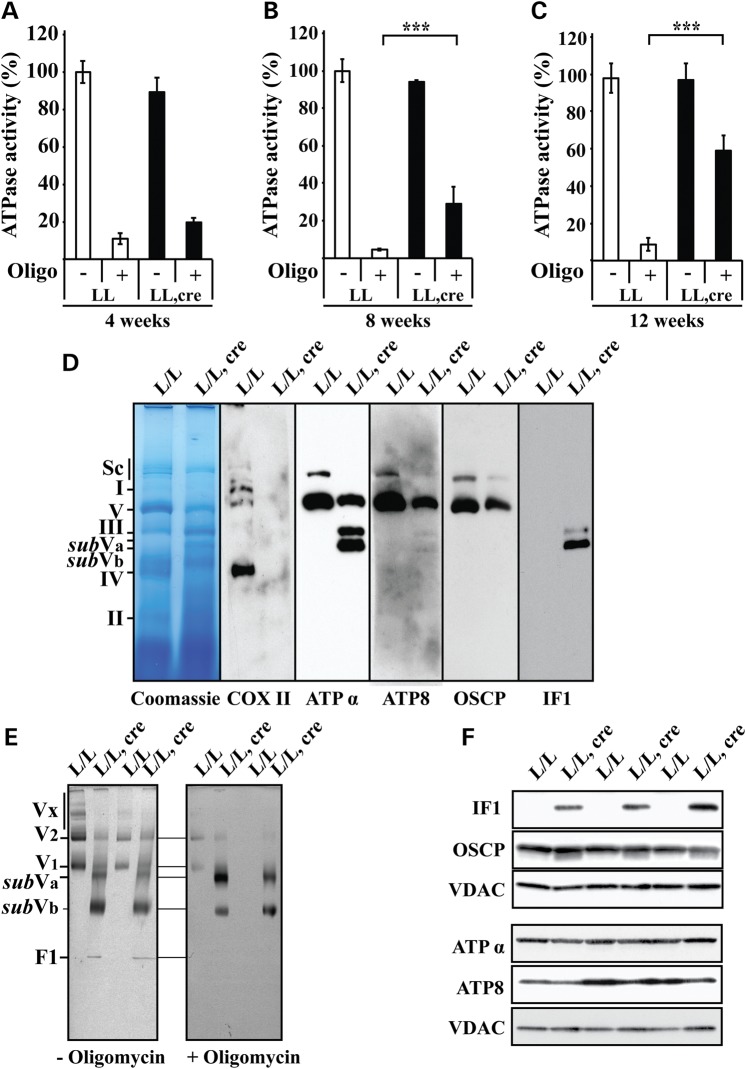
Loss of LRPPRC is associated with the appearance of an oligomycin-insensitive subassembled ATP synthase complex. (**A**) The ATPase activity in the presence or absence of oligomycin (Oligo) was normalized to the activity in controls measured in the absence of oligomycin, The ATPase activity was determined in heart mitochondria from *Lrpprc* knockout and control mice at age 4 weeks. Open bars, control (*n* = 3); filled bars, knockout (*n* = 3). Error bars indicate means ± SEM. (**B**) The ATPase activity in the presence or absence of oligomycin (Oligo) was normalized to the activity in controls measured in the absence of oligomycin. The ATPase activity was determined in heart mitochondria from *Lrpprc* knockout and control mice at the age of 8 weeks. Open bars, control (*n* = 3); filled bars, knockout (*n* = 3). Error bars indicate mean ± SEM (^*^P < 0.05; ^**^P < 0.01; ^***^P < 0.001). (**C**) The ATPase activity in the presence or absence of oligomycin (Oligo) was normalized to the activity in controls measured in the absence of oligomycin. The ATPase activity was determined in heart mitochondria from *Lrpprc* knockout and control mice at age 12 weeks. Open bars, control (*n* = 5); filled bars, knockout (*n* = 5). Error bars indicate means ± SEM (^*^P < 0.05; ^**^P < 0.01; ^***^P < 0.001). (**D**) Blue native polyacrylamide gel electrophoresis analysis of heart mitochondria extracted with a ratio (1 g/g) of digitonin to mitochondrial protein from *Lrpprc* knockout and control mice at the age of 12 weeks. Immunodetection of COX II, ATPα, ATP8, OSCP and IF1 after transfer of proteins from the BN-PAGE to PVDF membrane. The position of supercomplexes (Sc), complex I (I), the ATP synthase monomer (V), ATP synthase subcomplexes (*sub*Va, *sub*Vb), complex IV (IV) and complex II (II) are indicated on the left side. (**E**) The supra-molecular organization of ATP synthase was determined by clear native polyacrylamide gel electrophoresis (CN-PAGE) stained for in-gel ATPase activity (appear black after color inversion) without (right panel) or with oligomycin (left panel) in heart mitochondria from *Lrpprc* knockout and control at the age of 12 weeks. Mitochondria were solubilized with a ratio (1.5 g/g) of digitonin to mitochondrial protein (*n* = 6). The position of ATP synthase oligomers (Vx), dimer (V_2_), monomer (V_1_), subassemblies (*sub*Va, *sub*Vb) and F1 are indicated on the left side. (**F**) Steady-state levels of different ATP synthase subunits analyzed by western blots analyses in heart mitochondria from *Lrpprc* knockout and control mice at age 12 weeks (*n* = 3).

We proceeded to analyze the ATP synthesis system to further investigate the cause of the severe respiratory defect in the phosphorylating state in *Lrpprc* knockout heart mitochondria. The oligomycin-sensitive ATPase activity was analyzed as previously described (28) and we found an almost complete inhibition by oligomycin treatment of wild-type heart mitochondria (Fig. 3A–C). In contrast, the ATPase activity in *Lrpprc* knockout heart mitochondria at the age of 8 and 12 weeks was increasingly resistant to oligomycin (Fig. 3A–C). The observation that the total ATPase activity was normal, whereas the oligomycin-resistant activity was increased in *Lrpprc* knockout heart mitochondria (Fig. 3A–C) prompted us to analyze the assembly status of ATP synthase. To this end, we first determined that a ratio of 1 g/g digitonin to mitochondrial protein provided a good solubilization condition for characterization of the supra-molecular organization of ATP synthase by in-gel enzyme activity assays (Supplementary Material, Fig. S2A). Next, we proceeded to use blue native (BN-PAGE) (Fig. 3D, Supplementary Material, Fig. S2B) and clear native electrophoresis (CN-PAGE) (Fig. 3E, Supplementary Material, Fig. S2A and D) to determine the oligomerization state of the ATP synthase (29,30). As an internal control, we studied COX and found a substantial decrease of both the in-gel enzyme activity (Supplementary Material, Fig. S2C) and the levels of the assembled complex (Fig. 3D, Supplementary Material, Fig. S2B), consistent with the drastic reduction of COX enzyme activity in heart mitochondria of *Lrpprc* knockout mice (Fig. 1A). ATP synthase oligomers were present in heart mitochondria of *Lrpprc* conditional knockout mice at the age of 4 weeks (Supplementary Material, Fig. S2D), but these oligomers were almost totally lost in 12 weeks old conditional *Lrpprc* knockouts and instead sub-assembled complexes (here denoted s*ub*Va and s*ub*Vb) appeared (Fig. 3D and E, Supplementary Material, Fig. S2A and D). The CN-PAGE procedure allowed us to assess the oligomycin sensitivity of the different ATP synthase complexes (Fig. 3E). In control heart mitochondria, the in-gel ATPase activity was almost completely inhibited by oligomycin, whereas the *sub*Va and *sub*Vb subcomplexes in *Lrpprc* knockout heart mitochondria showed an ATPase activity that was resistant to oligomycin (Fig. 3E). To further characterize the basis for the oligomycin resistance in the subassembled ATP-synthase complexes, we investigated their subunit composition. The steady-state levels of the oligomycin sensitivity-conferring protein (OSCP) were not significantly altered on western blot analysis of heart mitochondria from conditional *Lrpprc* knockout mice at the age of 12 weeks (Fig. 3F). The level of ATPα was unchanged, whereas the level of ATP8 was reduced in total protein extracts form heart mitochondria of conditional *Lrpprc* knockout mice at the age of 12 weeks (Fig. 3F). Interestingly, despite normal IF1 mRNA levels (data not shown), the IF1 protein levels were dramatically increased in *Lrpprc* knockout heart mitochondria at the age of 12 weeks (Fig. 3F). Western blot analysis of ATP synthase complexes in heart mitochondria from conditional *Lrpprc* knockout mice at the age of 12 weeks showed that ATP8 and OSCP were mainly present in the assembled ATP synthase complex (Fig. 3D), ATPα was present in both the assembled complex and the subcomplexes (Fig. 3D), whereas IF1 was only found in the subcomplexes (Fig. 3D). As an independent, non-quantitative way to assess the protein composition of ATP synthase and subcomplexes, we performed mass spectrometry analysis (Table 1). We analyzed five samples of each complex and subcomplex and noted the number of times peptides from a particular protein subunit could be found (Table 1). The OSCP protein conferring oligomycin resistance was detectable in all ATP synthase complexes and subcomplexes (Table 1), however, western blot analysis of complexes separated by BN-PAGE revealed very low levels of the OSCP protein in the ATP synthase subcomplexes (Fig. 3D). These findings argue that most of the subcomplexes lack OSCP, which may explain their insensitivity to oligomycin. The IF1 protein was dramatically increased in *Lrpprc* knockout heart mitochondria (Fig. 3F) and was associated with the *sub*Va and *sub*Vb complexes (Fig. 3D). Western blot (Fig. 3D) and mass spectrometry (Table 1) analyses showed that most membranous domain (Fo) and catalytic domain (F1) subunits were present in the *sub*Va complex, whereas many of the Fo and F1 subunits as well as subunits of the stator domain were absent in the *sub*Vb complex. These results show that loss of LRPPRC leads to impaired ATP synthase assembly and the appearance of oligomycin-resistant ATP synthase subcomplexes.

**Table 1.** Subunit composition of dimers, monomers and subassembled ATP synthase complexes determined by mass spectrometry

Subunit	Mass (da)	PLGS score		Peptides		Coverage (%)		Incidence of detection (/5)						
								L/L		L/L, cre				
		L/L	L/L, cre	L/L	L/L, cre	L/L	L/L, cre	V2	V1	V2	V1	*sub*Va	*sub*Vb	F1
α	59 715	45 982	46 238	59	51	63	58	5	5	5	5	5	5	5
β	56 265	31 324	32 068	38	32	75	56	5	5	5	5	5	5	5
γ	16 751	8476	9171	14	10	36	34	5	5	5	5	5	2	4
δ	32 750	11 492	11 412	4	5	26	38	4	3	3	5	3	2	0
ɛ	5834	19 006	21 785	3	3	37	30	3	5	3	5	4	3	0
OSCP	23 348	14 405	20 730	18	13	68	60	5	5	5	5	5	5	3
b	28 930	3914	2688	9	7	33	24	5	5	4	5	4	1	0
e	8230	15 448	3020	5	6	47	53	5	5	5	5	3	2	4
f	8999	3516	4051	3	3	26	21	5	5	4	5	5	2	2
g	11 417	12 753	10 165	7	5	52	41	4	5	4	5	5	0	2
ATP8	7761	6763	8802	3	2	28	24	5	5	5	4	4	0	0

The ATP synthase dimer (V_2_), monomer (V_1_), subassembled complexes (*sub*Va and *sub*Vb) and F1 subunit composition in heart mitochondria from *Lrpprc* knockout and control mice at the age of 12 weeks (*n* = 5). The average PLGS score, number of peptides and coverage were calculated from values obtained in ATP synthase dimer (V_2_) and monomers (V_1_). The PLGS score was calculated by the Protein Lynx Global Server (PLGS 2.2.5) software and is a statistical measure of accuracy of assignation. A higher score implies greater confidence in protein identity. The incidence of detection in five replicates is presented in the right panel.

### Altered mitochondrial cristae morphology

**Figure 4.**
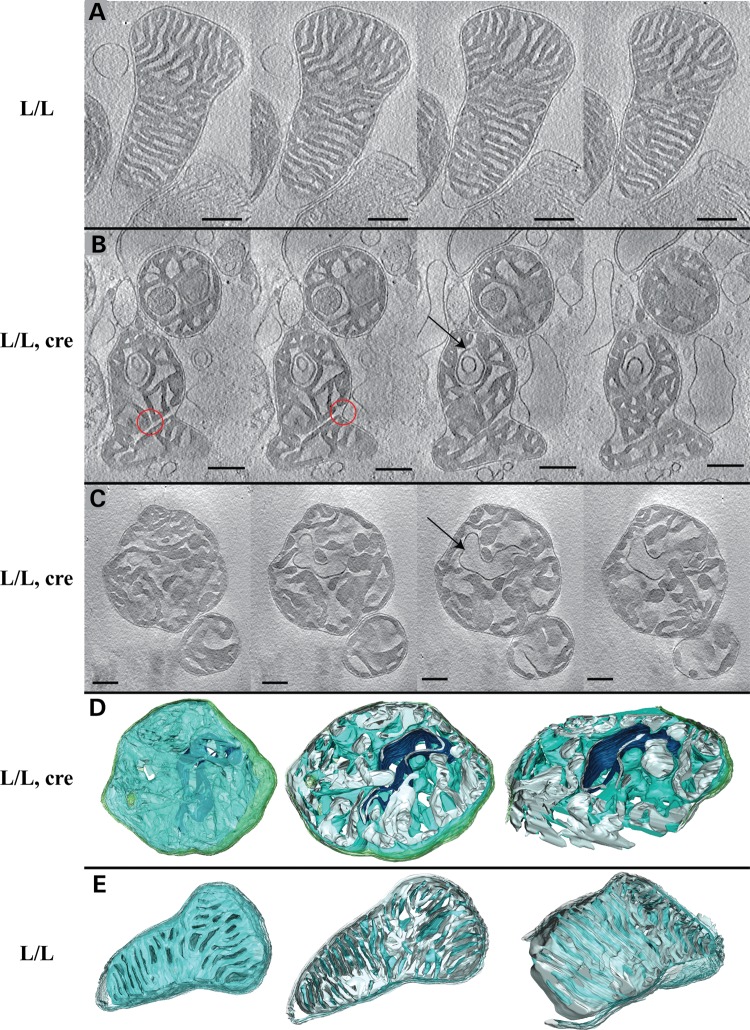
Loss of LRPPRC impairs mitochondrial cristae morphology. (**A**–**C**) Slices through tomograms of mitochondria analyzed by electron cryo-microscopy. Scale bars correspond to 250 nm. (A) An example of the highly organized cristae observed in heart mitochondria from control at the age of 12 weeks. Cristae are lamellar and form stacks. (B and C) Two examples of abnormal cristae morphology observed in heart mitochondria from *Lrpprc* knockout at the age of 12 weeks. The organization of the inner membrane with deeply invaginated cristae is lost. Instead, cristae are enlarged and the membrane curvature is partially inverted. The red circles indicate unusually large cristae junctions and connections between cristae. The arrows point towards dense, thin membrane compartments protruding from the matrix. (**D** and **E**) Three-dimensional segmentation of tomographic complete volumes (left) and cut-away views through the *xy*-plane (center) or an oblique slice (right). (D) Heart mitochondria from *Lrpprc* knockout mice at the age of 12 weeks (compare with C). (E) Heart mitochondria from control mice at the age of 12 weeks (compare with A). The membrane surface facing the cristae is shown in blue, the surface facing the matrix is shown in grey. The outer membrane is shown in transparent green. The dense, thin membrane compartment is shown in dark blue.

ATP synthase oligomers have recently been shown to cause membrane curvature by forming long dimer rows along the cristae ridges (31–35). The disassembly of ATP synthase dimer rows is accompanied by severe changes of cristae morphology with disappearance of the typical lamellar cristae in yeast mutants lacking dimer-specific subunits (35,36). Our previous electron microscopy studies using thin plastic sections of mouse heart tissue have shown altered cristae morphology in the absence of LRPPRC (20). Here, we further assessed mitochondrial morphology in isolated *Lrpprc* knockout heart mitochondria by performing electron cryo-tomography to obtain three-dimensional reconstructions of fully hydrated mitochondria at high resolution (Fig. 4 and Supplementary Material, Movies 1 and 2). Wild-type mitochondria from 12-week-old control mice had predominantly thin, stacked, lamellar cristae, with clearly discernible cristae junctions (Fig. 4A and E). In contrast, *Lrpprc* knockout heart mitochondria showed irregular inner membrane morphology (Fig. 4B–D). In many mitochondria, the shape and distribution of the cristae was irregular and lamellar cristae were absent (Fig. 4B and C). Cristae junctions were wider than in wild-type controls and in some cases not discernible. Furthermore, we observed several mitochondria in which canonical cristae were completely lost and the inner membrane instead formed a network of interconnected vesicles that extended throughout the mitochondrion (Fig. 4B–D). In comparison with regular cristae, the inner-membrane curvature was partially inverted and the characteristic cristae ridges typically occupied by ATP synthase (35) were largely missing. Occasionally, extremely dense and thin matrix compartments, essentially consisting of only two membranes, were observed.

Taken together, the abnormal cristae morphology (Fig. 4B–D), the reduced amount of oligomers of ATP synthase and the presence of ATP synthase subcomplexes (Fig. 3D and E) in *Lrpprc* knockout heart mitochondria support previous reports (36,32) that ATP synthase oligomerization is required for proper cristae organization.

### Bioenergetic consequences of ATP synthase subassembly on membrane potential and ROS production

**Figure 5.**
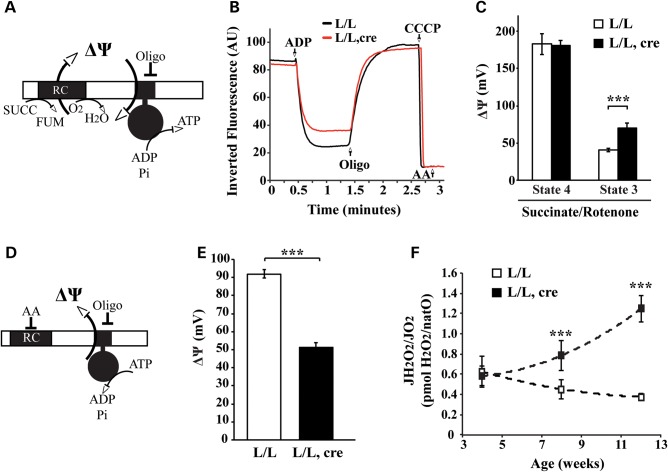
Subassembled ATP synthase fails to translocate protons and increases ROS production. (**A**) Scheme of the conditions used to measure the membrane potential (ΔΨ) in isolated mitochondria incubated with succinate and rotenone under phosphorylating conditions. (**B**) Inverted rhodamine 123 fluorescent signal recording in heart mitochondria from *Lrpprc* knockout (red line) and control (black line) mice at the age of 12 weeks. Fluorescent signal variation was followed after successive addition of ADP, oligomycin (Oligo), CCCP and Antimycin A (AA). (**C**) Quantification of the membrane potential (ΔΨ) under non-phosphorylating (State 4) and phosphorylating (State 3) conditions in heart mitochondria from *Lrpprc* knockout and control mice at the age of 12 weeks. Open bars, control (*n* = 4); filled bars, knockout (*n* = 4). Error bars indicate means ± SEM (^*^P < 0.05; ^**^P < 0.01; ^***^P < 0.001). (**D**) Scheme of the conditions used to measure the membrane potential (ΔΨ) in isolated mitochondria incubated with ATP and antimycin A. These conditions allow the ATP synthase to generate membrane potential (ΔΨ) by hydrolyzing ATP. (**E**) The mitochondrial membrane potential imposed by the ATPase in heart mitochondria from *Lrpprc* knockout and control mice at age 12 weeks. Open bars, control (*n* = 4); filled bars, knockout (*n* = 4). Error bars indicate means ± SEM (^*^P < 0.05; ^**^P < 0.01; ^***^P < 0.001). (**F**) The mitochondrial peroxidic yield, i.e. hydrogen peroxide released per oxygen consumed, was assessed under phosphorylating conditions with succinate and rotenone in heart mitochondria from *Lrpprc* knockout and control at different ages. Open square, control (*n* = 4); filled square, knockout (*n* = 4). Error bars indicate means ± SEM (^*^P < 0.05; ^**^P < 0.01; ^***^P < 0.001).

The presence of oligomycin-insensitive, partially assembled ATP synthase complexes in *Lrpprc* knockout heart mitochondria prompted us to further investigate the bioenergetic properties. Under physiological conditions, ATP synthase couples the membrane potential (ΔΨ) consumption to ATP synthesis. We measured the ΔΨ under phosphorylating and non-phosphorylating conditions (Fig. 5A–C). In agreement with other models harboring a specific ATP synthase deficiency (37–39), mitochondria from *Lrpprc* knockout hearts can maintain ΔΨ in the non-phosphorylating state, but are hyperpolarized under phosphorylating conditions (Fig. 5B and C, Supplementary Material, Fig. S3A). Consequently, the decrease in ATP production in *Lrpprc* knockout heart mitochondria (Fig. 1C) is associated with decreased ΔΨ consumption. We proceeded to investigate the ability of ATP synthase to couple proton transfer to ATP usage by assaying the enzyme activity under ATP hydrolyzing conditions, thus uncoupling its enzyme activity from the function of the RC (Fig. 5D). It is well known that specific pH conditions can release inhibitory peptides (IF1) from the ATP synthase (40,41) and thereby allow the generation of an increased ΔΨ by hydrolysis of ATP (Fig. 5D). Our results show that the ATP synthase in *Lrpprc* knockout heart mitochondria cannot maintain a high ΔΨ when ATP is hydrolyzed (Fig. 5E). Next, we assessed hydrogen peroxide release when the oxidative phosphorylation system is working under phosphorylating conditions (Fig. 5F). The hydrogen peroxide production per oxygen consumed, a.k.a*.* peroxidic yield (42), was dramatically increased in *Lrpprc* knockout heart mitochondria at the ages of 8 and 12 weeks (Fig. 5F, Supplementary Material, Fig. S3B). Our data are in accordance with previous observations from studies on patient cell lines that have an ATP synthase deficiency because of a pathogenic mutation in the ATP6 gene of mtDNA (43–45). The ATP synthase defect in these patient cell lines causes a mitochondrial hyperpolarization and an increase in reactive oxygen species (ROS) production. Altogether, the results presented here strongly argue that the partly assembled ATP synthase complexes in *Lrpprc* knockout heart mitochondria cannot properly couple ATP synthesis or hydrolysis to proton translocation across the inner membrane. Interestingly, the observed hyperpolarization under phosphorylating conditions (Fig. 5C) is associated with an increased ROS production (Fig. 5F). However, this increased ROS production is not associated with increased levels of protein and lipid carbonylation (Supplementary Material, Fig. S3C), or with an increase in the steady-state levels of mitochondrial superoxide dismutase (SOD2) (Supplementary Material, Fig. S3D).

## DISCUSSION

The role of LRPPRC in mammalian mitochondria has attracted much interest as mutations in *Lrpprc* cause LSFC, a severe progressive form of infantile onset neurodegeneration associated with COX deficiency. Recent advances in our understanding of the molecular mode of action of LRPPRC based on studies in knockout mice (20) and knockdown flies (26) have demonstrated that LRPPRC is a key posttranscriptional regulator of mtDNA expression that is necessary for mRNA stability, mRNA polyadenylation and coordination of mitochondrial translation. Despite this progress, the bioenergetic consequences associated with loss of LRPPRC have not been fully elucidated. LSFC patients have a profound COX deficiency and this defect has been considered to be of key importance for explaining the disease pathophysiology. However, the global decrease in mtDNA expression makes it possible that there are other OXPHOS problems, besides the COX deficiency, that are driving the phenotype. To investigate this hypothesis, we carried out an extensive bioenergetic and morphological characterization of mitochondria lacking LRPPRC. Surprisingly, we report here that the main consequence of loss of LRPPRC is deficient ATP production due to direct effects on ATP synthase oligomerization and assembly. In contrast, COX deficiency is not the main cause of the OXPHOS dysfunction.

The bioenergetic characterization of *Lrpprc* knockout heart mitochondria shows that only a small fraction, <10%, of the COX enzyme activity is required to maintain OXPHOS under phosphorylating conditions. This large excess in COX capacity, also defined as COX reserve capacity, has been described in different tissues and models, but remains poorly understood (46–48). Our results indicate that the substantial COX reserve capacity explains why the *Surf1* knockout mouse is largely asymptomatic (14–16). Moreover, the fact that severe myocardial dysfunction cannot be detected in COXVIaH knockout hearts, despite ∼80% reduction in COX activity (17), supports our conclusion that cardiac mitochondria possess a substantial COX capacity reserve.

In contrast to previous models, we show here that loss of *Lrpprc* in heart does not only impair COX activity but it also affects the ATP synthase complex organization and activity. The electron cryo-tomography analysis of *Lrpprc* knockout heart mitochondria shows that the loss of ATP synthase oligomers has a severe effect on cristae morphology. Additionally, we show here that the ATP synthase subcomplexes are oligomycin-resistant and fail to properly couple ATP hydrolysis to proton translocation. So far the OSCP protein has been described as key to confer oligomycin sensitivity to the ATP synthase. Recent structural analyses has shown that OSCP links the α subunit of the catalytic part (F1) to the subunit b of the stator (49). This stator-mediated physical connection between the F1 and Fo domains seems essential for inhibiting ATP synthesis or hydrolysis in the presence of oligomycin. The lack of oligomycin-induced inhibition of the ATP synthase subcomplexes can be due to the very low levels of OSCP or lack of the oligomycin-binding sites in subunits c (50) and ATP6 (51–53). The ATP synthase regulatory peptide IF1 has been shown to have a major role on ATP synthase function and structure (54). The IF1 peptide binds the catalytic part of the ATP synthase to prevent futile ATP hydrolysis. Interestingly, the increased level of IF1 and its association with the ATP synthase subcomplexes could be a safeguarding mechanism that explains the absence of ATP hydrolysis under physiological pH conditions in *Lrpprc* knockout heart mitochondria. The presence of the ATP synthase subcomplexes does not affect the inner membrane proton conductance and does not uncouple the OXPHOS despite impairing the overall ATP production capacity.

Patient cell lines with pathogenic mutations of the ATP synthase subunit 6 (ATP6) (55,56) or subunit 8 (ATP8) genes (57) of mtDNA show defects in the supra-molecular organization of ATP synthase with appearance of ATP synthase subcomplexes (58). A similar type of ATP synthase destabilization has previously been reported in mouse models with impaired mtDNA expression due to mtDNA replication defects (59), impaired mtDNA transcription or impaired mitochondrial translation (60–62). The presence of the ATP synthase subcomplexes, therefore, seems to constitute a sensitive marker for the loss of mtDNA expression.

The severe ATP synthase deficiency reported here sheds important new light on the bioenergetic consequences of LRPPRC loss of function mutants. The ATP synthase activity is rarely measured when investigating OXPHOS defects and has not been investigated in LSFC patient tissue samples (22,63) or fibroblast cultures (25). LSFC patients have a different spectrum of associated symptoms compared with other types of COX-deficient LS and often develop an acute fatal acidosis crisis. Our finding that loss of LRPPRC does not only affect COX activity, but also impairs the ATP synthase may provide an important explanation for the clinical difference between LSFC and forms of LS caused by isolated COX deficiency.

## MATERIALS AND METHODS

### Mouse breeding

Breeding and genotyping of heart- and skeletal muscle-specific *Lrpprc* knockout mice were performed as previously described in a C57Bl6/N background (20). Briefly, *Lrpprc*^loxP/loxP^ mice were crossed with transgenic mice expressing cre recombinase under the control of the muscle creatine kinase promoter (Ckmm-cre). The resulting double heterozygous mice (*Lrpprc*^loxP/+,^ +/Ckmm-cre) were mated to *Lrpprc*^loxP/loxP^ mice to generate tissue-specific knockout (*Lrpprc*^loxP/loxP^, +/Ckmm-cre) and control (Lrpprc^loxP/loxP^) mice. The Surf1 mice were obtained from *Surf1*^+/−^ intercrosses. Surf1 mice were analyzed at 40 weeks of age.

### Mitochondrial isolation from heart

Mice were sacrificed by cervical dislocation, and hearts were quickly collected in ice-cold DPBS (Gibco), minced and homogenized with few strokes of a Potter S homogenizer (Sartorius) in 5 ml of ice-cold mitochondria isolation buffer (MIB; 310 mm sucrose, 20 mm Tris–HCl, 1 mm EGTA, pH 7.2). Mitochondria were purified by differential centrifugation (1200*g* for 10 min) and supernatants were then centrifuged at 12 000*g* for 10 min. The crude mitochondrial pellet was resuspended in an appropriate volume of MIB. The mitochondrial protein concentration was determined using the Protein DC Lawry-based assay (Bio-Rad).

### Mitochondrial respiratory assay

Mitochondrial oxygen consumption flux was measured as previously described (64) at 37°C using 65–125 μg of crude mitochondria diluted in 2.1 ml of mitochondrial respiration buffer (120 mm sucrose, 50 mm KCl, 20 mm Tris–HCl, 4 mm KH_2_PO_4_, 2 mm MgCl_2_, 1 mm EGTA, pH 7.2) in an Oxygraph-2k (OROBOROS INSTRUMENTS, Innsbruck, Austria). The oxygen consumption rate was measured using either 10 mm pyruvate, 5 mm glutamate and 5 mm malate or 10 mm succinate and 10 nm rotenone. Oxygen consumption was assessed in the phosphorylating state with 1 mm ADP (state 3) or non-phosphorylating state by adding 2.5 μg/ml oligomycin (pseudo state 4). In the control mitochondria, the respiratory control ratio (RCR) values were >10 with pyruvate/glutamate/malate and >5 with succinate/rotenone. Respiration was uncoupled by successive addition of carbonyl cyanide m-chlorophenyl hydrazone (CCCP) up to 3 μM to reach maximal respiration.

### Threshold curve of COX control on mitochondrial respiration in phosphorylating condition

As previously described (48), the titration of respiration in the phosphorylating condition with a COX-specific inhibitor (KCN) allows the assessment of the control exerted by this enzyme on the OXPHOS function. In this experiment, we defined the variation of the respiration in phosphorylating condition induced by a decrease in the COX enzyme activity. The mitochondrial respiration was measured with succinate 10 mm and rotenone 10 nm or with pyruvate 10 mm, glutamate 5 mm, malate 5 mm and ADP 1 mm, as described above, in presence of different concentrations of KCN (up to 4 mm). The KCN titration of the COX activity was assessed by measuring the oxygen consumption rate of mitochondria incubated with the same KCN concentration and with TMPD 0.2 mm, ascorbate 1 mm and antimycin A 0.5 µM as described below. The titration curve of the COX activity by KCN in control and *Lrpprc* knockout heart mitochondria is presented in Supplementary Material, Figure S1C.

### Measurement of ATP synthesis flux (JATP)

Isolated mitochondria (65 µg/ml) were suspended in the mitochondrial respiration buffer (see above). After addition of ADP (1 mm), succinate (2 mm) and rotenone (10 nM) or addition of ADP (1 mm), pyruvate (10 mm), glutamate (5 mm) and malate (5 mm), the oxygen consumption and ATP synthesis rates were both measured. Aliquots were collected every 20 s and precipitated in 7% HClO_4_/25 mm EDTA, centrifuged at 16 000 g for 10 min and then neutralized with KOH 2 M, MOPS 0.3 M. The ATP content in these samples was determined with the ATPlite 1step from PerkinElmer^®^. In a parallel experiment, oligomycin (2.5 μg/ml protein) was added to the mitochondrial suspension to determine the non-oxidative ATP synthesis rate.

### Measurement of isolated enzyme activity (COX and ATPase)

The COX activity was assessed using a classical TMPD/ascorbate assay. Briefly, isolated mitochondria (65 µg/ml) were suspended in mitochondrial respiration buffer (see above). Oxygen consumption was assessed in the presence of TMPD (0.2 mm), ascorbate (1 mm) and antimycin A (0.5 µM). After few minutes of stationary respiration, KCN (2 mm) was injected into the chamber. The COX activity corresponds to the KCN sensitive respiration. ATPase activity was assessed as previously described (65). Shortly, 65 µg/ml freshly isolated mitochondria were incubated at 37°C in the following buffer: triethanolamine 75 mm, MgCl_2_ 2 mm, pH 8.9. Mitochondria were preincubated 2 min with alamethicin 10 µg/ml. The reaction was started by adding 2 mm of ATP. Samples (150 µl) were removed every 2 min and precipitated in 7% HClO_4_/25 mm EDTA (50 µl). Phosphate was quantified by incubating 150 µl of each aliquot in 1 ml of the following buffer (molybdate 5.34 mm, ferrous sulfate 28.8 mm, and H_2_SO_4_ 0.75 N). After 2 min incubation, the absorbance was assessed at 600 nm. In a parallel experiment, oligomycin (2.5 μg/ml protein) was added to the mitochondrial suspension to determine the oligomycin-insensitive ATPase activity.

### Measurement of reactive oxygen species

The rate of H_2_O_2_ production was determined by monitoring the oxidation of the fluorogenic indicator amplex red in the presence of horseradish peroxidase. The concentrations of horseradish peroxidase and amplex red in the incubation medium were 5 U/ml and 1 µM, respectively. Fluorescence was recorded at the following wavelengths: excitation 560 nm and emission 590 nm. A standard curve was obtained by adding known amounts of H_2_O_2_ to the assay medium in the presence of the reactants. Mitochondria (65 µg protein/ml) were incubated in the respiratory medium (see above), at 37°C and the H_2_O_2_ production rate measurement was initiated by substrate addition. The H_2_O_2_ production rate was determined from the slope of a plot of the fluorogenic indicator versus time.

### Membrane potential measurement

Trans-membrane potential variations (ΔΨ) in isolated mitochondria were assayed by monitoring the fluorescence quenching of rhodamine 123 with a Hitachi F7000 fluorimeter. The ΔΨ was estimated from the fluorescence quenching of the lipophilic cationic dye rhodamine 123. Isolated mitochondria (65 µg protein/ml) were incubated in the mitochondrial buffer thermostated at 37°C containing succinate (10 mm) or pyruvate (10 mm), glutamate (5 mm), malate (5 mm) and 0.66 µM of rhodamine 123 (Sigma). When added, ADP was 1 mm, ATP 2 mm, oligomycin 2.5 µg/ml.

The rhodamine fluorescence signal at each steady state (F) was recorded using an excitation wavelength of 485 nm. Fluorescence emission was continuously detected at 500 nm. At the end of each experiment, the maximum fluorescence signal (Fmax) was monitored after complete de-energization of the mitochondria following addition of CCCP (6 µm) and antimycin A (0.5 µm). Then, the Fmax-F/Fmax difference at each steady state was calculated.

The membrane potential imposed by the ATPase activity was assessed in the following buffer (155 mm sucrose, 10 mm Tris–HCl, 0.5 mm EGTA, triethanolamine 37.5 mm, MgCl_2_ 1 mm, pH 8.2). After 3 min incubation of the mitochondria in 2 ml of buffer containing pyruvate, glutamate, malate, we added antimycin A (0.5 µg/ml) and ATP 2 mm and the oligomycin-sensitive ΔΨ was recorded.

### Clear native PAGE and in gel activity

Clear native PAGE was performed as previously described (66,42). Briefly, 0.1 mg of frozen isolated mitochondria (never thawed) were suspended in 1% digitonin solubilized in the following buffer [potassium acetate 150 mm, HEPES 30 mm, glycerol 12%, 6-aminocaproic acid 2 mm, EDTA 1 mm and 1 pastille of protease inhibitor mini complete (ROCHE), pH 7.4]. Samples were incubated 30 min on ice and centrifuged at 24 000*g* for 30 min. The supernatant was then supplemented with 6-aminocaproic acid (final concentration: 37.5 mm) and loaded on CN-PAGE (3–13%). Samples migrated overnight at 100–120 V. The gel was incubated at 37°C for 1 h, in the following buffer (triethanolamine 75 mm, MgCl_2_ 5 mm, 0.1% triton X100, lead acetate 0.5 mg/ml, pH 8.9) to obtain the ATPase in gel activity. To detect the oligomycin-insensitive complex, oligomycin (50 µg/ml) was added. The densitometry analysis was performed with the free software FIJI.

### Blue native electrophoresis and immunodetection

Blue native electrophoresis (BN-PAGE) was performed in 4–16% gradient gels according to recommendation of the Novex^®^ NativePAGE™ Bis-Tris gel System. Nitrocellulose membranes were used to transfer proteins from SDS gels and PVDF membranes were used to transfer proteins from native gels. Immunodetection of ATP5A1 (Complex V) was performed with specific monoclonal antibodies (Mitoscience). Immunodetection of IF1 was performed using a monoclonal antibody (ab110277; Mitosciences). Immunodetection of OSCP was performed using a polyclonal antibody (sc-74786; Santacruz). VDAC (porin) antibodies were purchased from Calbiochem and polyclonal antisera were used to detect COXII (COX) and ATP8 (67). Protein carbonylation assays were performed according to the Oxyblot protein oxidation kit instructions from Millipore and using the HNE polyclonal antibody from Abcam (ab46544). Immunodetection of SOD2 was performed using a polyclonal antibody from Millipore (AB10346).

### Mass spectrometry analysis

The ATP synthase localized by in gel activity bands were excised from the CN-PAGE, chopped into small cubes and transferred to the wells of an OASIS^®^ HLB µElution Plate (Waters Corporation, Milford, CT, USA). The proteins were digested with trypsin (Gold MS grade, Promega, Madison, WI, USA) in an OASIS^®^ plate subsequently (68). Protein identification was performed with a Xevo Q-Tof (Waters Corporation) coupled with a nanoACQUITY UPLC^™^ (Waters Corporation); 1–4 µl of tryptic digest was directly loaded into an analytical column of 75 µm × 150 mm C18 BEH 1.7 µm (Waters Corporation) with 1% formic acid for 25 min. The loading flow rate was 400 nl/min. The peptides were eluted with a gradient of 3–55% acetonitrile in 0.1% formic acid over 90 min at a flow rate of 400 nl/min. The Xevo Qtof (Waters Corporation) was operated in LC/MS^E^ mode over the m/z range 50–1800 in the Nano-Electrospray mode. The capillary, sample cone, extraction cone and collision energy were 3.6 kV, 25.0, 2.0 and 6.0 V, respectively. During elevated energy scan, the collision energy was ramped from 15 V to 35 V. Glu-fibrinopeptide B of m/z 785.84 was used as Lock Mass for mass correction. Data were collected using MassLynx^™^ 4.1 and processed using ProteinLynx^™^ Global Server 2.4. The following are the parameters for database search: minimal fragments ion per peptide matched 3, minimal fragments ion per protein matched 7, missed cleavages 1, fixed modification: carbamidomethyl cysteine; variable modifications: acetyl N-term, oxidation methione.

### Mitochondrial electron cryo-tomography

For analysis by electron cryo-microscopy, mitochondria were washed twice with 320 mm trehalose, 20 mm Tris pH 7.3, 1 mm EGTA. Samples were mixed 1:1 with fiducial gold markers (10 nm gold particles conjugated to protein A, Aurion), blotted and immediately plunge-frozen in liquid ethane on Quantifoil holey carbon grids (Quantifoil Micro Tools). Single tilt series (±60°, step size 1.5°) were collected on a FEI Polara (300 kV) using an Ultrascan 4 × 4 k CCD (Gatan) and a post-column Quantum energy filter (Gatan) at −9 µm defocus. The nominal magnification was ×34 000, resulting in a pixel size of 6 Å. A total dose of ∼130 e^−^/Å² was used. Tilt series alignment using the gold fiducial markers and tomogram reconstruction by back-projection were carried out using the IMOD software package (69). To increase contrast, a final filtering step applying non-linear anisotropic diffusion (70) was performed. Manual segmentation was performed with the program Amira (Mercury Systems).

## SUPPLEMENTARY MATERIAL

Supplementary Material is available at *HMG* online.

## FUNDING

The study was supported by ERC Advanced Investigator, Cluster of Excellence CECAD and Deutsche Forschungsgemeinschaft, SFB 829, grants to N.G.L. Funding to pay the Open Access publication charges for this article was provided by the Max Planck Society.

## Supplementary Material

Supplementary Data
