# Supplementary material for: Loss of LRPPRC causes ATP synthase deficiency
Source: Hum Mol Genet. 2014 Jan 6;23(10):2580–92. doi: 10.1093/hmg/ddt652 (PMC3990160; doi:10.1093/hmg/ddt652)
Supplement: Supplementary Data [file supp_23_10_2580__index.html]

Loss of LRPPRC causes ATP synthase deficiency — Loss of LRPPRC causes ATP synthase deficiency — Supplementary Data 

# Loss of LRPPRC causes ATP synthase deficiency

## Supplementary Data

Supplementary Data

**Files in this Data Supplement:**

- Supplementary Data - Doc file
- Supplementary Video 1 - mpg file
- Supplementary Video 2 - mpg file
