# Supplementary material for: Loss of LRPPRC causes ATP synthase deficiency
Source: Hum Mol Genet. 2014 Jan 6;23(10):2580–92. doi: 10.1093/hmg/ddt652 (PMC3990160; doi:10.1093/hmg/ddt652)

**SUPPLEMENTAL DATA**

**Figure legends**

**Figure S1.** Assessment of ATP synthesis and COX activity inhibition threshold curve by using complex I substrates (**A**) ATP synthesis flux assessed in heart mitochondria from *Lrpprc* knockout and control at age 12 weeks in the presence of pyruvate, glutamate, malate and ADP. Open bars, control (n = 5); filled bars, knockout (n = 5). Error bars indicate mean ± SEM (^*^p<0.05; ^**^p<0.01; ^***^p<0.001). (**B**) COX inhibition threshold curve performed in heart mitochondria from *Lrpprc* knockout and control mice at age 12 weeks in the presence of pyruvate, glutamate, malate and ADP. Open circles, controls (n = 3), filled circles, knockouts (n = 3) (**C**) Representation of the COX inhibition threshold curve in relation to the KCN concentration. The inhibition curve was determined in heart mitochondria from *Lrpprc* knockout and control mice at age 12 weeks in the presence of TMPD, ascorbate and ADP (circles) or TMPD, ascorbate and CCCP (squares). Open symbols and the continuous line indicate controls (n = 6); filled symbols and the dashed line indicate knockouts (n = 6).

**Figure S2.** Loss of LRPPRC is associated with the appearance of sub-assembled ATP synthase complexes. (**A**) Colorless native polyacrylamide gel electrophoresis (CN-PAGE) of heart mitochondria from *Lrpprc* knockout and control mice at age 12 weeks. The heart mitochondria were extracted with different ratios of digitonin to mitochondrial protein. A sequential ATPase in gel activity and Coomassie staining are performed for each condition (n = 3). (**B**) Blue native gel electrophoresis analysis performed with digitonin or DDM treated heart mitochondria from *Lrpprc* knockout mice at ages 4 and 12 weeks (n = 3). (**C**) Blue native polyacrylamide gel electrophoresis and CN-PAGE analyses combined with in gel COX activity (IGA) to determine COX supramolecular organization in *Lrpprc* knockout and control mice at age 12 weeks. Mitochondria were extracted with 1 g/g of digitonin to mitochondrial protein. BN-PAGE and CN-PAGE gels were loaded with the same amount of extracted protein (n = 3). (**D**) CN-PAGE performed with heart mitochondria from *Lrpprc* knockout and control mice at ages 4 and 12 weeks. Mitochondria were extracted with 1 g/g of digitonin to mitochondrial protein. The densitometry analysis of the CN-PAGE of the *Lrpprc* knockout (red line) and control mitochondria (black line) is presented on the right side of each gel (n = 3).

**Figure S3.** Loss of LRPPRC is associated with OXPHOS dysfunction but does not increase the protein and lipid carbonylation levels. (**A**) Quantification of the membrane potential (ΔΨ) under non-phosphorylating (State 4) and phosphorylating (State 3) conditions in heart mitochondria from *Lrpprc* knockout and control mice at age 12 weeks. Open bars, controls (n = 4); filled bars, knockouts (n = 4). Error bars indicate mean ± SEM (^*^p<0.05; ^**^p<0.01; ^***^p<0.001). **(B)** Mitochondrial peroxidic yield, *i.e* hydrogen peroxide released per oxygen consumed, assessed under phosphorylating conditions with pyruvate, glutamate, malate, and ADP in heart mitochondria from *Lrpprc* knockout and control mice at age 12 weeks. Open bars indicate controls (n = 4) and filled bars indicate knockouts (n = 4). Error bars indicate mean ± SEM (^*^p<0.05; ^**^p<0.01; ^***^p<0.001). **(C**) Oxyblot and western blot analysis of HNE in heart mitochondria from *Lrpprc* knockout and control mice at age 12 weeks (n = 3). **(D**) SOD2 protein levels analyzed by western blots of heart mitochondria from *Lrpprc* knockout and control mice at age 12 weeks. (n = 3)

**Supplementary Video 1.** Tomographic volume of a control heart mitochondrion and 3D segmentation. Note the highly organized stacks of lamellar cristae. The membrane surface facing the cristae is shown in blue and the surface facing the matrix is shown in grey. The outer membrane is shown in transparent grey.

**Supplementary Video 2.** Tomographic volume of a *Lrpprc* knockout heart mitochondrion and 3D segmentation. The organization of the inner membrane into deeply invaginated cristae is lost. Cristae are enlarged and the membrane curvature is partially inverted. The membrane surface facing the cristae is shown in blue, the surface facing the matrix is shown in grey. The dense, thin membrane compartment is shown in dark blue. The outer membrane is shown in transparent green.


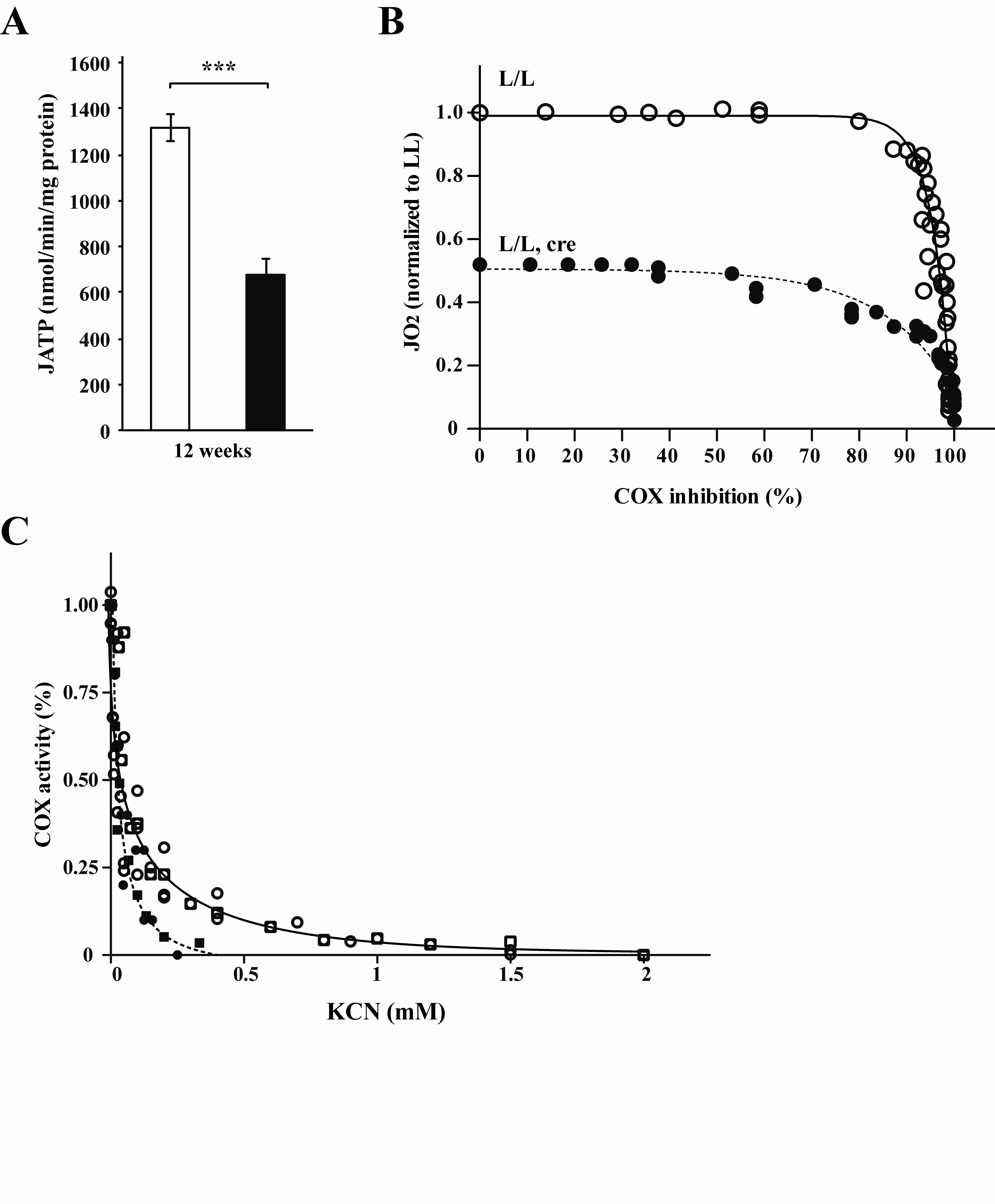


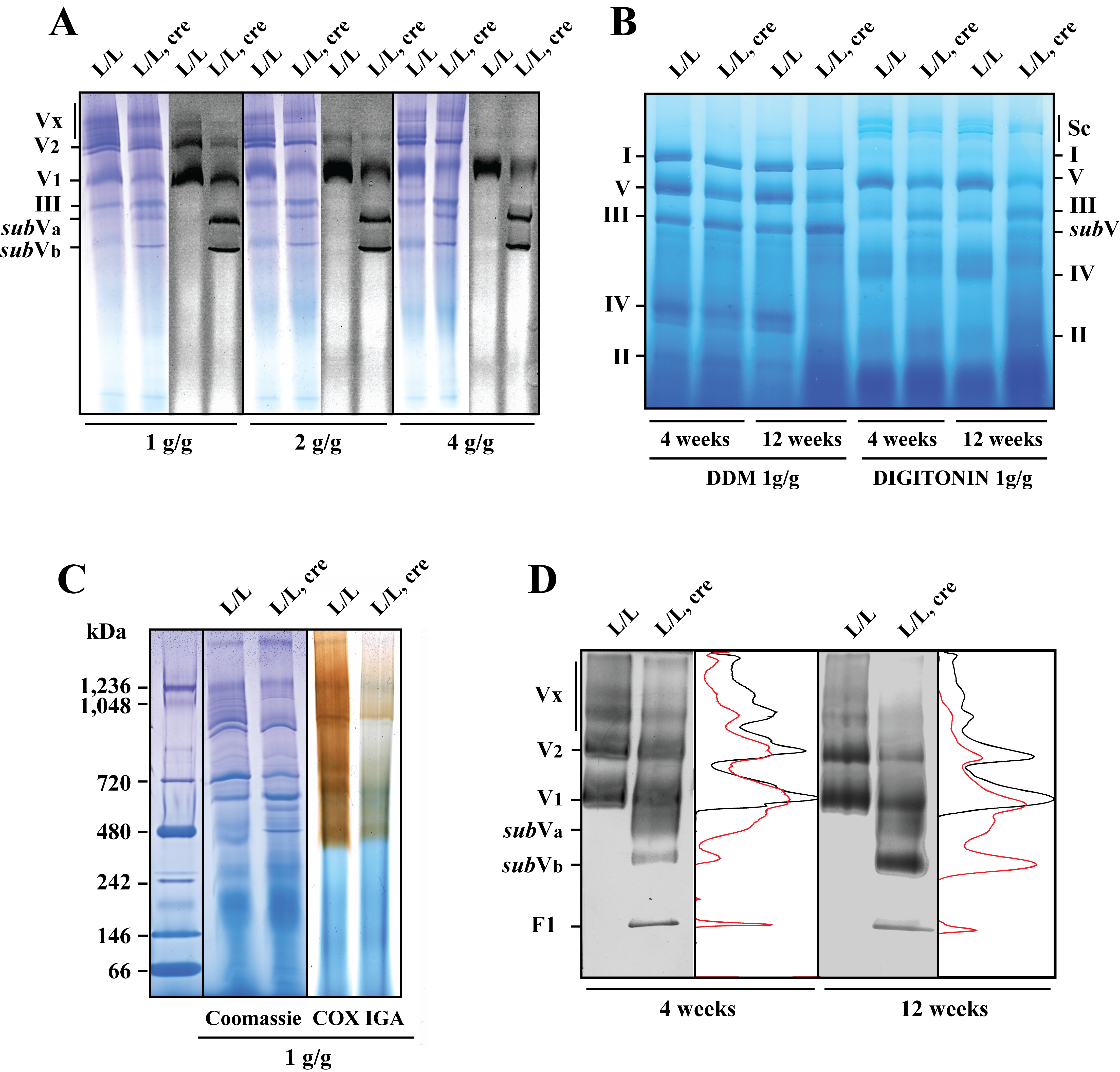


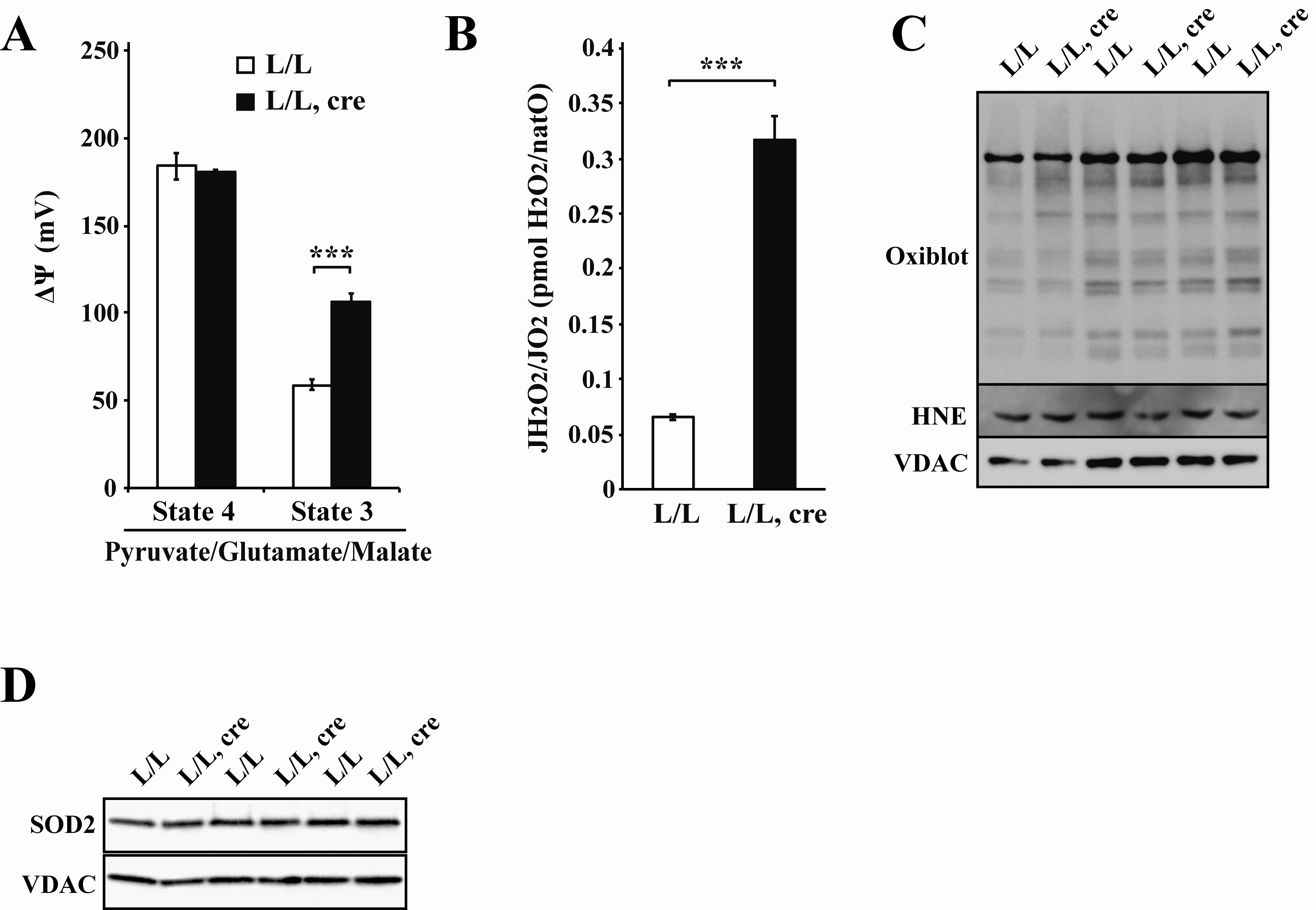

Supplement: Supplementary Data [file supp_ddt652_ddt652supp.doc]
